# Modeling and biomechanical characterization of femur and tibia bones using the Extended Mooney–Rivlin approach with mathematical validation

**DOI:** 10.1016/j.jor.2025.06.035

**Published:** 2025-07-01

**Authors:** Mohamed Hassan, A.S. Abdel-Rahman

**Affiliations:** a Department of Engineering, School of Computing and Engineering, University of Huddersfield, United Kingdom; b Physics Department, Faculty of Science, Cairo University, Giza, Egypt

**Keywords:** Human bony leg, Extended Mooney-Rivlin model, Elastic modulus, Shear modulus, Internal friction, Femur, Tibia, Finite element analysis

## Abstract

**Background:**

Understanding the non-linear mechanical behavior of human bone is critical for improving orthopedic modeling and developing personalized treatment strategies. The Mooney-Rivlin model, traditionally used in soft matters, has been extended to capture the complex stress–strain relationships of hard biological materials like bone.

**Objective:**

To apply the Extended Mooney-Rivlin model to human bone specimens and quantify regional variations in mechanical parameters, with the goal of improving finite element simulations and biomechanical interpretations.

**Participants and setting:**

The study analyzed bone specimens from the proximal femur as well as the midshaft, distal, and proximal sections of long bones in the lower limb, based on data obtained from the literature.

**Methods:**

Experimental stress–strain data were collected from bone samples subjected to uniaxial loading. The Extended Mooney-Rivlin model was fitted to the data to extract four key parameters: *B* (overall stiffness), *C*_*1*_ (shear resistance), *C*_*2*_ (damping/energy dissipation), and *H* (non-linearity).

**Results:**

The model demonstrated strong goodness-of-fit across all specimens (*R*^*2*^ > 0.95). Stiffness (*B*) was significantly higher in midshaft regions compared to distal regions. Damping capacity (*C*_*2*_) and linearity (*H*) were elevated in distal regions *C*_*2*_, indicating enhanced shock-absorbing properties. Surprisingly, shear resistance (*C*_*1*_) was also greater in trabecular-rich regions, reflecting greater adaptability to complex loading environments.

**Conclusions:**

The Extended Mooney-Rivlin model effectively captures regional variations in bone mechanics, with clear distinctions between cortical and trabecular bone behavior. These findings support its application in advanced biomechanical modeling and suggest new directions for personalized orthopedic treatment. Future work should explore the influence of age, bone mineral density, and pathological changes on these mechanical parameters.

## Abstract (author highlights)

The novelty of the featured article.

➢ Introduces a more accurate and physiologically representative modeling approach for human lower limb bone mechanics, surpassing traditional finite element methods in fitting precision.
➢ Provides detailed mapping of mechanical properties across anatomical regions of the femur and tibia.
➢ Offers a comprehensive explanation of regional variations in elastic modulus, shear modulus, and internal friction (damping factor) for femoral and tibial bone tissues.
➢ Presents an engineering-based perspective on failure mechanisms in human lower limb bones, offering valuable insights for biomechanical analysis, orthopedic implant design, and injury prevention.

➢ Introduces a more accurate and physiologically representative modeling approach for human lower limb bone mechanics, surpassing traditional finite element methods in fitting precision.
➢ Provides detailed mapping of mechanical properties across anatomical regions of the femur and tibia.
➢ Offers a comprehensive explanation of regional variations in elastic modulus, shear modulus, and internal friction (damping factor) for femoral and tibial bone tissues.
➢ Presents an engineering-based perspective on failure mechanisms in human lower limb bones, offering valuable insights for biomechanical analysis, orthopedic implant design, and injury prevention.

## 1 Introduction

Understanding the mechanical behavior of bone is essential for enhancing clinical decision-making, designing orthopedic implants, and developing accurate computer models for injury prevention and rehabilitation.^1^ Bone consists of approximately 60–70 % minerals (such as calcium and phosphate), 25–30 % water, and collagen. The minerals provide compressive strength, while collagen contributes to bone's flexibility.^2^

Bone exhibits both anisotropic and viscoelastic properties. Anisotropy means that its mechanical characteristics—such as stiffness and strength—vary depending on the direction of the applied force. This behavior arises from bone's internal organization, where collagen fibers and mineral crystals are aligned in specific directions. Consequently, bone responds differently to loads applied in different orientations. Viscoelasticity refers to bone's combination of immediate elastic response and time-dependent deformation under load, which can lead to a gradual reduction in stiffness over time.^3^

At the nanoscale, collagen and hydroxyapatite interact to form mineralized collagen fibrils. These fibrils are further assembled into hierarchical structures, such as lamellae and osteons, particularly in cortical bone. This complex hierarchical organization gives bone its distinctive nonlinear mechanical behavior, meaning its response to stress is not directly proportional to the applied load.^4^

Recent studies have underscored the limitations of traditional hyperplastic models in accurately simulating the complex, nonlinear mechanical behavior of bone tissue. The Neo–Hookean model was found to be inadequate for capturing the nonlinear responses observed in experimental data. Stress–strain curves from compression tests revealed pronounced nonlinearity, characterized by low stiffness at small strains and significant stiffening at higher strain levels. Consequently, the Neo–Hookean model significantly underestimated the tissue response at higher strains, resulting in a poor overall fit—evidenced by low coefficients of determination (mean R^2^ = 0.831) and elevated root mean square error (RMSE) values when compared to more sophisticated models.^5^

Also, more advanced hyperelastic formulations, such as Ogden-type models—particularly the 4-term Ogden and the combined Logarithmic–Ogden models—are theoretically better suited to describe the nonlinear behavior of soft tissues. These models incorporate multiple parameters and exponents, enabling them to account for the material's complex mechanical response. However, in practical application, these models often encounter parameter convergence issues during the curve-fitting process, limiting their reliability and robustness.^5^

The Mooney–Rivlin hyperelastic model demonstrated good agreement with experimental results obtained from microindentation tests on human trabecular bone. When comparing simulated force–displacement curves to experimental data, the model accurately replicated both the shape and peak values of the loading and unloading phases. Quantitatively, the difference in hysteresis loop area between experimental and simulated results remained below 15 % across all trials, and discrepancies in maximum indentation depth were also within a 15 % margin. These findings indicate that the Mooney–Rivlin model is capable of effectively capturing the nonlinear elastic response of trabecular bone under quasi-static loading conditions.^6^

However, minor deviations were noted, particularly in the post-unloading phase. These inconsistencies may be attributed to the biological variability of trabecular bone architecture and the limitations of modeling the tissue as an isotropic material, which may not fully capture its anisotropic nature.^6^

Neo-Hookean materials exhibit nonlinear behavior under large stress, producing greater strain than predicted by the linear elastic region (Hookean region), eventually leading to plastic deformation and failure. The Mooney–Rivlin model effectively captures this nonlinear response, with its formulation derived from kinetic theory. Although the model accounts for volume changes during deformation, its accuracy is primarily limited to the small strain range, also known as before the Gaussian region.^7^, ^8^, ^9^, ^10^, ^11^

However, during the deformation of composites, the energy generated from interactions between molecular chains varies with the average intermolecular distance. In other words, composite deformation involves not only volumetric changes but also energy changes. In solids, internal friction can be defined as the energy dissipation associated with deviations from Hooke's law. As such, the magnitude of internal friction effectively reflects the change in internal energy during deformation. Consequently, it is essential to employ a theoretical stress–strain model to analyze internal friction variations in different composites. The Mooney–Rivlin model is a widely used constitutive model for various materials; however, it is typically valid only for strains up to approximately 100 % in tension and 30 % in compression. Moreover, it neglects the influence of internal friction on the mechanical behavior of composites.^12^ To address these issues, **Abdel-Rahman** proposed a semi-empirical modification to the basic Mooney–Rivlin equation by incorporating the effects of internal friction.^13^

To address Mooney–Rivlin modeling limitation, an extended Mooney–Rivlin model^13^ is introduced. This model captures the full stress–strain behavior, from the initial elastic region through to large deformations. It is based on a theoretical derivation involving a critical elongation value, a concept observed in prior studies but not previously defined from a theoretical perspective.

Additionally, **Miura** et al.^14^ developed a subject-specific, CT-based finite element analysis (FEA) model of the proximal femur using fine tetrahedral elements and validated it through mechanical testing with cadaveric samples. Their findings revealed that commonly used material models, such as those proposed by Keyak, significantly overestimated bone stiffness, in some cases by as much as a factor of ten. Even Keller's equations, which were comparatively more accurate, still overpredicted mechanical values by approximately 1.6 times. These results suggest that existing material models may be inadequate for accurately simulating the mechanical behavior of bone tissue.

**Filardi** and **Milardi**^15^ investigated the mechanical behavior of the lower body under vertical loading using a combination of experimental testing and finite element (FE) modeling. Strain measurements were obtained from six strain gauges placed on key regions of the femur and tibia. When compared to the FE-predicted strain values, the results showed an average relative error of 25.33 %, indicating a notable discrepancy. This level of error suggests that the FE model did not accurately capture the mechanical behavior of the lower limb.

Therefore, this study aims to characterize and compare the nonlinear mechanical properties—such as elastic modulus, shear modulus, internal friction, and the linearity of the stress–strain relationship before the cut-off point—of three lower limb bone segments. The goal is to enhance the fidelity of computational simulations and contribute to a deeper biomechanical understanding of bone behavior under functional loads.

Previous studies using finite element analysis (FEA) have employed various modeling approaches, yet many have failed to achieve a close fit between experimental data and theoretical predictions. In this investigation, an extended Mooney–Rivlin model was applied to human leg bones. The resulting mechanical parameters can serve as a detailed map of the leg's structural behavior, with potential applications in orthopedics,^16^, ^17^, ^18^, ^19^ mechanical design,^20^, ^21^, ^22^, ^23^, ^24^ material properties' analysis,^25^, ^26^, ^27^, ^28^, ^29^, ^30^, ^31^, ^32^, ^33^, ^34^ and various other physical^35^, ^36^, ^37^, ^38^, ^39^ and engineering contexts.^40^, ^41^, ^42^, ^43^

## 2 Methods

**Fig. 1:**
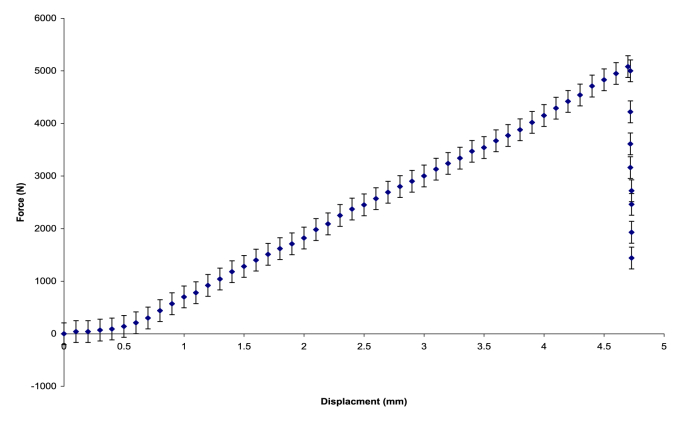
Force–displacement curve digitized from **Miura** et al.,^14^ representing the mechanical behavior of the proximal femur under compression testing using cadaveric specimens.

Experimental data were extracted from published graphs to assess the mechanical behavior of the lower limb bones. Specifically, the force–displacement curve presented by **Miura,**^14^ which represents the mechanical testing of cadaveric proximal femur samples, was digitized and analyzed. Microsoft Excel's Goal seek and curve-fitting tools were employed to retrieve the underlying data points (Fig. 1). This dataset reflects the mechanical response of the proximal femur under compressive loading, serving as a reference for subsequent model validation.

**Fig. 2:**
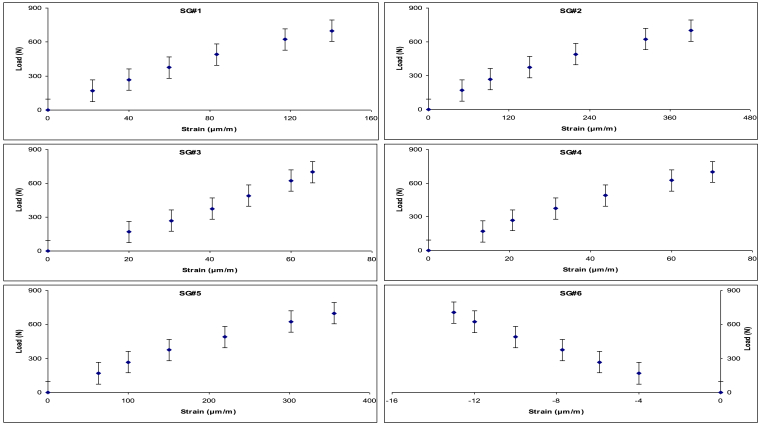
Experimental Load–starin curves retrieved from **Filardi** and **Milardi**^15^ for six reference points along the femur and tibia (proximal, middle, and distal regions).

Similarly, six force–displacement curves from the study by **Filardi** and **Milard**i^15^ were also digitized and analyzed using the same Excel-based method. These curves correspond to experimental mechanical tests conducted at six specific reference points, where strain gauge sensors (SGS) were positioned along the proximal, middle, and distal regions of both the femur and tibia (Fig. 2). This dataset provides localized insights into the mechanical response of different segments of the lower limb under vertical loading.

Once the experimental curves were retrieved, the data were modeled using the Extended Mooney–Rivlin hyperelastic model, enabling a more accurate representation of the nonlinear mechanical behavior observed at each anatomical site in both studies. This advanced model yielded improved fitting results and provided deeper insight into the elastic characteristics of the femoral and tibial bone segments under loading.

The Extended Mooney–Rivlin model was applied to fit the extracted experimental data and to characterize the nonlinear elastic response of bone tissue. The general form of the model, as referenced in Ref. 13, is presented in Equation (1), which relates the Mooney stress (*σ*_*Mooney*_) to the elongation ratio (*λ*). The model includes four fitting parameters - B, C_1_, C_2_, and H - which were determined through curve-fitting procedures based on the experimental data.

(1) $$\sigma_{Mooney}=\frac{\sigma_{true}}{\left(\lambda -\frac{1}{\lambda^{2}}\right)}=B\frac{\lambda_{C}-\lambda }{\lambda -1}+C_{2}+C_{1}\lambda +H\lambda^{2}$$

where *λ*_*C*_ is the critical elongation constant, with a value of 1.4655.^13^ The true stress (*σ*_*true*_) is calculated from the applied load (*F*) and the affected cross-sectional area (*A*), as defined by the following equation:

(2) $$\sigma_{true}=\frac{F}{A}\lambda$$

To facilitate analysis, equation (1) can be reformulated in terms of a modified stress variable, referred to as the Extended Mooney Stress (*σ*_*Ext.Mooney*_), as shown in Equation (3).

(3) $$\sigma_{Ext.Mooney}=\frac{\sigma_{true}\left(\lambda -1\right)}{\left(\lambda -\frac{1}{\lambda^{2}}\right)}=\frac{\sigma_{true}\left(\lambda -1\right)\lambda^{2}}{\left(\lambda^{3}-1\right)}=\frac{\sigma_{true}\left(\lambda -1\right)\lambda^{2}}{\left(\lambda -1\right)\left(\lambda^{2}+\lambda +1\right)}=\frac{\sigma_{true}\lambda^{2}}{\left(\lambda^{2}+\lambda +1\right)}$$

This formulation allows Equation (3) to be expressed as a polynomial function of the elongation ratio *λ*, as shown in Equation (4).

(4) $$\sigma_{Ext.Mooney}=\left[B\lambda_{C}-C_{2}\right]+\lambda \left[C_{2}-C_{1}-B\right]+\lambda^{2}\left[C_{1}-H\right]+\lambda^{3}\left[H\right]$$

Alternatively, it can be expressed in terms of strain (*ε* = *λ*-1), as shown in Equation (5). This formulation enables the curve to originate at zero, in contrast to the elongation-based expression, which begins at unity.

(5) $$\sigma_{Ext.Mooney}=\left[B\left(\lambda_{C}-1\right)\right]+\varepsilon \left[C_{2}+C_{1}+H-B\right]+\varepsilon^{2}\left[C_{1}+2H\right]+\varepsilon^{3}\left[H\right]$$

Using the fitted load–strain curves and the derived equations, the coefficients of the Extended Mooney–Rivlin model were calculated. Each coefficient in the model carries a distinct physical interpretation. The parameter *B* is directly associated with the elastic modulus and is always strictly positive, reflecting the material's inherent stiffness. The coefficient *C*_*1*_ is related to the shear modulus, while *C*_*2*_ represents internal friction, capturing the material's damping characteristics. The parameter *H* describes the material's behavior under high-stress conditions, particularly near failure or specimen cut-off. All coefficients, except for *B*, may take either positive or negative values, depending on the mechanical response of the tissue.^13^

The third-order polynomial regression fit of Equation (5) using the least squares method may yield a negative strain-independent (*ε*-free) term, which corresponds to the *B*(*λ*_*C*_−1) component. While a negative *B* coefficient may provide the best possible mathematical fit—minimizing the sum of squared residuals, it is scientifically unacceptable, as *B* represents a physical quantity (elastic modulus) that must remain strictly positive.

To resolve this issue, Equation (5) can be extrapolated to a region near zero strain, allowing the curve to intercept the Extended Mooney stress axis, from which a positive estimate of *B* can be inferred. Alternatively, to retain *B* as an unfitted constant, a new fitting parameter *D* may be introduced, as follows:

(6) $$D=\frac{\left(\sigma_{Ext.Mooney}-B\left(\lambda_{C}-1\right)\right)}{\varepsilon }=\left[C_{2}+C_{1}+H-B\right]+\varepsilon \left[C_{1}+2H\right]+\varepsilon^{2}\left[H\right]$$

The new fitting approach employs a second-order polynomial regression, which allows for the estimation of the *C*_*1*_, *C*_*2*_, and *H* coefficients, while keeping *B* as a fixed parameter.

## 3 Results

**Fig. 3:**
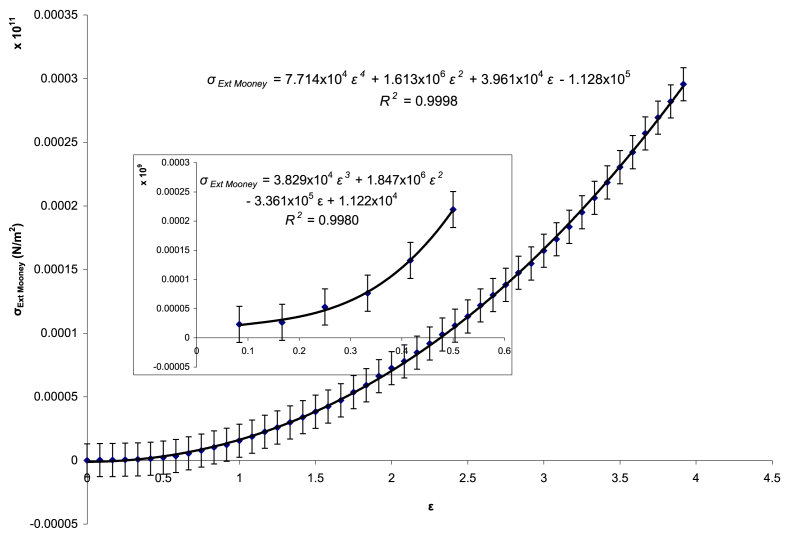
Extended Mooney stress–strain curves with third-order polynomial regression fitting.

The data from Fig. 1 were re-plotted as Extended Mooney stress (*σ*_*Ext.Mooney*_) versus strain (*ε*) and are presented in Fig. 3, accompanied by third-order polynomial regression curves obtained through the least squares fitting method.

The third-order polynomial regression fitting shows a negative *ε*-free term, leading to a negative value for the coefficient ***B***, which is physically inadmissible. The inset in Fig. 3 provides a magnified view of the initial portion of the curve, up to a strain of 0.5. In this range, the *ε*-free term is positive, resulting in *B* = 2.4104 × 10^4^ N/m^2^. By re-plotting parameter *D* as a function of strain (*ε*), a second-order polynomial regression was obtained. The corresponding regression coefficients and R^2^ values are presented in Table 1.

**Table 1:** Fitting the coefficients of the Extended Mooney–Rivlin model based on previous mechanical testing of specimens.

Coefficient	Value	Unit
*B*	2.410 × 10^4^	N/m^2^
*C*_*1*_	1.770 × 10^6^	
*C*_*2*_	−2.120 × 10^6^	
*H*	3.829 × 10^4^	
*R*^*2*^	0.9986	–

**Fig. 4:**
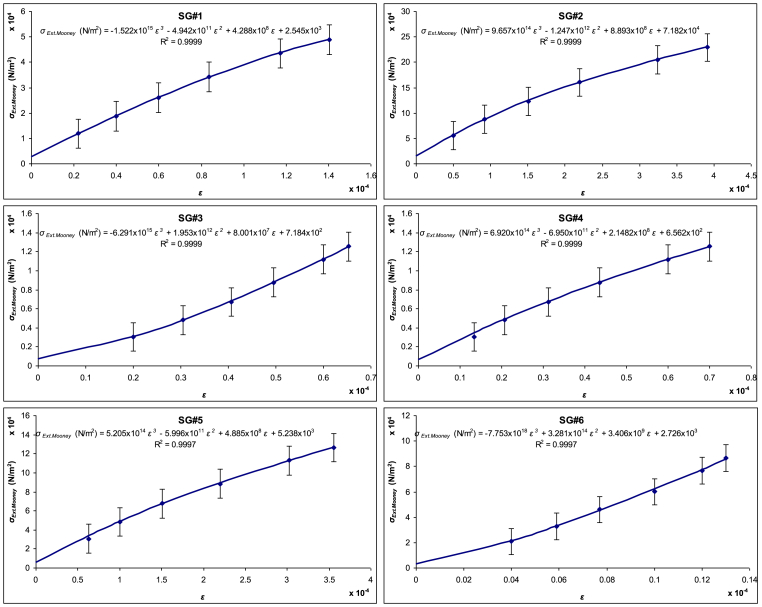
Extended Mooney stress–strain curves for six SGS reference points along the femur and tibia, with third-order polynomial regression fitting.

The data from Fig. 2 were re-plotted as Extended Mooney stress (*σ*_*Ext.Mooney*_) versus strain (*ε*) and are presented in Fig. 4. This figure includes six force–displacement curves corresponding to strain gauge sensor (SGS) locations at the proximal, middle, and distal regions of both the femur and tibia. Third-order polynomial regression lines, fitted using the least squares method, are also shown for each curve.

**Fig. 5:**
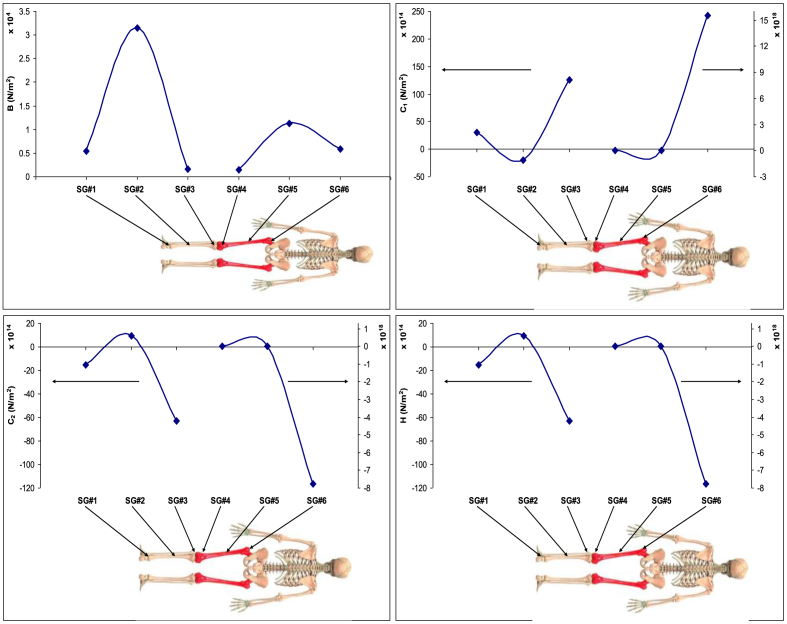
Mapping of Extended Mooney-Rivlin coefficients (*B*, *C*_*1*_, *C*_*2*_, and *H*) across the entire bony leg, highlighting the spatial distribution of each coefficient.

Subsequently, the distribution of the coefficients *B*, *C*_*1*_, *C*_*2*_, and *H* was mapped across the nodes of the bony leg, as illustrated in Fig. 5. Each coefficient corresponds to distinct mechanical properties, enabling an analysis of their spatial variation in relation to bone function and structural characteristics.

The *B* values were highest in the middle region (SG#2 and SG#5), indicating that the diaphyseal part of the femur is the stiffest and most resistant to axial deformation. In contrast, the proximal and distal regions exhibited lower *B* values, reflecting reduced stiffness consistent with the greater presence of trabecular bone in these areas.

The shear modulus coefficient, associated with *C*_*1*_, showed a gradual increase from the distal to proximal regions. The proximal femur (SG#6) demonstrated the highest shear resistance, while the distal and middle regions exhibited comparatively lower values, indicating regional variations in load-bearing adaptation.

The *C*_*2*_ values—which reflect the amount of energy dissipated through internal friction—were highest at SG#3 and SG#6, suggesting that the distal region has the greatest damping capacity. This implies a higher ability to absorb shock and reduce transmitted forces, aligning with the structural characteristics of trabecular bone. In contrast, the middle gauges (SG#2 and SG#5) had lower *C*_*2*_ values, indicating reduced internal friction and higher mechanical efficiency.

The high-stress behavior parameter (*H*) followed a trend similar to *C*_*2*_. The middle region (SG#2 and SG#5) showed greater mechanical stability under elevated loading conditions, while the distal region (SG#3 and SG#6) had the lowest stability, as evidenced by their lower *H* values. These findings support the concept of regional mechanical specialization along the femur, balancing stiffness, damping, and load-bearing functions.

## 4 Discussion

Understanding the mechanical properties of bones is essential for enhancing clinical decision-making, optimizing orthopedic implant design, and developing accurate computational models for injury prevention and rehabilitation. In this study, the Extended Mooney–Rivlin model was employed to investigate the biomechanical characteristics of the femur and tibia. Experimental data were collected from previously published studies^14^^,^^15^ to evaluate the mechanical behavior of these lower limb bones. The nonlinear response of bone tissue was effectively captured using the Extended Mooney–Rivlin model, which allowed for accurate fitting of the experimental data and characterization of the bone's elastic behavior. Through this approach, key model parameters -B, C_1_, C_2_, and H- were identified, providing valuable insights into the material properties governing bone mechanics.

The findings of the current study revealed that *B* values, which represent the elastic modulus, were highest in the midshaft regions of both the femur and tibia, and lowest in the proximal regions, a trend that aligns with previous research. This pattern is primarily attributed to the predominance of cortical bone in the diaphyseal (midshaft) areas. As reported by **Osterhoff** et al.,^44^ cortical bone is significantly stiffer and capable of withstanding higher ultimate stresses than trabecular bone, although it exhibits greater brittleness. Its relatively homogeneous biomechanical behavior contributes to the greater stiffness and strength observed in the mid-diaphyseal regions.

Additionally, **Bousson** et al.^45^ have shown that the mid-diaphyseal region of the femur exhibits a high cortical bone mineral density (cBMD), which is associated with low porosity and enhanced mechanical integrity. This region contains a markedly higher cortex-to-trabecular bone volume ratio compared to the metaphyseal (end) regions, reflecting an adaptation to meet the mechanical demands of load-bearing and force transmission. In contrast, the proximal femur is characterized by a higher proportion of trabecular bone, which although less stiff than cortical bone, plays a vital role in stress distribution and mechanical support, particularly in regions subject to complex, multi-directional loading conditions.^46^

Furthermore, **Cuppone** et al.^47^ reported that the longitudinal Young's modulus in the femoral mid-shaft can reach 18,600 ± 1900 MPa, indicating a high resistance to deformation under physiological loading. This elevated stiffness is biomechanically significant, as the diaphysis region, composed predominantly of dense cortical bone, is adapted to withstand substantial compressive and bending forces encountered during weight-bearing and locomotor activities.

In contrast, the proximal region of the femur, including the femoral neck and head, exhibits a lower elastic modulus due to its higher content of trabecular bone, which is structurally adapted for energy absorption and load distribution. **Lotz** et al.^48^ reported an average elastic modulus of 9650 ± 2410 MPa in the proximal metaphyseal region, approximately 24 % lower than that of the mid-diaphysis. This reduction in stiffness reflects the region's biomechanical adaptation to buffer impact forces transmitted to the hip joint during gait and other dynamic activities. Supporting this, **Blain** et al.^49^ demonstrated that cortical bone thickness progressively decreases toward the proximal femur, accompanied by a greater proportion of trabecular bone, particularly within the femoral neck.

The distal region of the femur shows a similar pattern. **Huppke** et al.^50^ assessed cortical thickness in the distal femur and observed that it is thinner compared to the midshaft, resulting in lower stiffness values. This structural configuration is well-suited for distributing load across the knee joint, while also preserving a degree of shock absorption capacity, which is essential for joint protection during dynamic loading.

On the other hand, **Tsurumoto** et al.^51^ analyzed femoral cortical bone and confirmed that the mid-diaphyseal region possesses a substantial cortical layer, which contributes significantly to the mechanical strength and load-bearing capacity of the femur. Moreover, these studies emphasize the regional variation in elastic modulus along the femur. This variation reflects an optimized balance between mechanical stiffness and functional demands tailored to the specific biomechanical roles of different anatomical regions.

The shear modulus (*G*) exhibits a linear relationship with the coefficient *C*_*1*_.^13^ It is evident that the shear modulus is higher for trabecular bone (distal), where the loading axis may deviate or slope. This indicates that trabecular bones are more rigid and less susceptible to deformation under significant stress, whereas the cortical bone (midshaft) regions are comparatively more deformable. The central shaft of the leg bones can endure axial loading with minimal transverse deformation, often expressed as a slight curvature of the bone.

Notably, the femur shows a higher shear modulus than the tibia, particularly for trabecular bones. This characteristic is functionally appropriate for the fovea capitis femoris, which operates under a broader angular range compared to the medial condyle of the tibia.^52^

The structural design of leg bones is primarily optimized to support axial body loads. In the event of a lateral fall, where the body impacts the ground from the side, kinetic energy is rapidly transformed into potential energy and manifests as shear stress within the bones. This shear stress tends to concentrate in regions with the lowest shear moduli, where fractures are more likely to occur. These fractures act as energy absorbers, helping to prevent damage in regions of higher shear modulus.

Fractures occurring in the midshaft regoins are generally less critical than those for trabecular bone (distal), where immediate surgical intervention is often required to protect adjacent cartilage structures. This mechanism exemplifies the concept of failure guidance engineering, in which shear stresses exceeding structural limits are intentionally directed toward less critical regions—such as midshafts—minimizing the risk of severe damage at vital load-bearing or articulating distal.^53^

The damping coefficient, which exhibits a linear dependence on the coefficient *C*_*2*_, was found to be highest at the distal region of the femur and tibia. This suggests that these regions are most effective in dissipating mechanical energy, thereby exhibiting the greatest internal friction. In contrast, the middle regions demonstrated lower *C*_*2*_ values, indicative of reduced internal friction and greater mechanical efficiency in force transmission. These findings are consistent with the distinct mechanical roles of cortical and trabecular bone.

Trabecular bone, more abundant in the distal regions, possesses a porous, honeycomb-like structure that enhances its ability to absorb shock and vibrations. This structural configuration helps reduce the transmission of impact forces, leading to increased energy dissipation during loading conditions.^54^ Conversely, the cortical bone, which predominates in the midshaft regions, is denser and more compact. This density makes it less effective at damping vibrations, allowing it to transmit mechanical forces more directly and efficiently along the bone's length.^55^ While trabecular bone is designed for shock absorption and energy dissipation, it is less capable of sustaining high stresses without deformation. In contrast, cortical bone offers greater mechanical stability and load-bearing capacity, albeit with lower energy dissipation capability.

The coefficient *H* characterizes the deviation of the stress–strain curve from linear behavior prior to the yield or cut-off point. The analysis revealed that the linearity of the stress–strain relationship is more pronounced for the trabecular bone (distal) than for cortical bone (midshaft). This observation can be attributed to the higher shear moduli in these regions, where loads are more likely to be transferred obliquely along the bone axis. The increased linearity in these areas plays a crucial role in maintaining bone geometry and preventing deformation, which is essential for preserving the functional integrity of joint mechanisms, particularly in hinge-like structures such as the knee and hip.^56^

## 5 Conclusion

This study highlighted the critical importance of understanding the mechanical behavior of human bone, especially in the context of biomedical simulation, implant design, and orthopedic surgical planning. By employing the Extended Mooney-Rivlin hyperelastic model, we successfully simulated and analyzed the nonlinear stress-strain behavior of bone tissue under physiologically realistic loading conditions. The study focused on the femur and tibia, examining mechanical responses across different anatomical regions to capture spatial variability in material behavior.

The findings revealed that the parameter *B*, representing the elastic modulus, was highest in the midshaft (diaphyseal) regions of both the femur and tibia and lowest in the proximal regions. This distribution is consistent with prior studies and reflects the anatomical variation in bone composition. The midshaft regions are dominated by cortical bone, characterized by a dense, low-porosity structure that provides superior stiffness and load-bearing capacity. This structural organization allows cortical bone to effectively resist deformation under axial and bending loads. Conversely, the proximal regions, which contain a higher proportion of trabecular bone, exhibit reduced stiffness but are biomechanically adapted for energy absorption and multidirectional stress distribution, particularly under complex joint loading conditions.

The parameter *C*_*1*_, associated with shear deformation and initial stiffness, was found to be higher in trabecular bone, particularly in the distal regions of the femur and tibia. This suggests that the trabecular architecture provides enhanced resistance to multidirectional shear forces—an essential characteristic in anatomically complex regions such as the femoral head, which experiences a wide range of motion. Notably, although cortical bone exhibits greater overall stiffness, it is more prone to deformation under oblique or off-axis loading conditions. This anisotropic behavior influences the stress transfer and distribution patterns along the bone shaft, underscoring the functional specialization of cortical and trabecular bone in responding to region-specific mechanical demands.

The damping parameter *C*_*2*_, indicative of internal friction and energy dissipation capacity, was highest in the distal regions of both the femur and tibia. This corresponds to the spongy, porous architecture of trabecular bone, which is well-suited for absorbing mechanical shocks and protecting joints during impact. Such structural adaptation enhances the bone's ability to dissipate kinetic energy effectively. In contrast, the midshaft regions exhibited lower *C*_*2*_ values, reflecting the predominance of dense cortical bone that transmits axial loads more efficiently. These regions are structurally optimized for force conduction rather than energy absorption, consistent with their biomechanical role in supporting body weight and facilitating locomotion.

The non-linearity parameter *H* quantified the extent of deviation from linear stress–strain behavior, which was more pronounced in cortical bone, particularly at higher strain levels. This behavior reflects the stiff and brittle nature of cortical bone under elevated loading conditions. In contrast, trabecular bone demonstrated a more linear stress–strain response prior to yielding, suggesting a capacity to maintain joint alignment and function under oblique or multidirectional loads. This distinction underscores the biomechanical specialization of bone regions: cortical bone is optimized for structural integrity and load-bearing, while trabecular bone contributes to flexibility, energy absorption, and protection of articulating joints.

Overall, the Extended Mooney–Rivlin model effectively captured the regional mechanical behavior of the human femur and tibia. The observed variation in model parameters highlights the adaptive structural organization of bone tissue, tailored to meet distinct functional and loading requirements across anatomical regions. These findings enhance the accuracy of finite element simulations in orthopedic biomechanics and provide valuable insights for the design of patient-specific implants, personalized surgical planning, strategies for fracture prevention and bone repair, and contribute to more accurate and predictive computational models in orthopedics.

## Consent statement

Not applicable. This study did not involve human participants or patient data requiring consent.

## Ethics statement

Ethical approval was not required for this work as it did not involve human subjects or biological samples.

Clinical trial number: not applicable.

## Credit author statment

All authors have contributed equally to the conception, design, Formal analysis, and writing of this study. All authors have reviewed and approved the final version of the manuscript.

## Ethiacal statement

This study did not involve human or animal subjects and therefore did not require ethical approval.

## Funding statement

This research did not receive any specific grant from funding agencies in the public, commercial, or not-for-profit sectors.

## Declaration of competing interest

Authors declare no conflict of interest.
